# *Lactobacillus gasseri* SBT2055 Reduces Infection by and Colonization of *Campylobacter jejuni*

**DOI:** 10.1371/journal.pone.0108827

**Published:** 2014-09-29

**Authors:** Keita Nishiyama, Yasuyuki Seto, Kazuki Yoshioka, Tsutomu Kakuda, Shinji Takai, Yuji Yamamoto, Takao Mukai

**Affiliations:** 1 Department of Animal Science, School of Veterinary Medicine, Kitasato University, Towada, Aomori, Japan; 2 Milk Science Research Institute, Megmilk Snow Brand Co. Ltd., Kawagoe, Saitama, Japan; 3 Faculty of Veterinary Medicine, School of Veterinary Medicine, Kitasato University, Towada, Aomori, Japan; Charité-University Medicine Berlin, Germany

## Abstract

*Campylobacter* is a normal inhabitant of the chicken gut. Pathogenic infection with this organism in humans is accompanied by severe inflammation of the intestinal mucosal surface. The aim of this study was to evaluate the ability of *Lactobacillus gasseri* SBT2055 (LG2055) to inhibit the adhesion and invasion of *Campylobacter jejuni in vitro* and to suppress *C. jejuni* colonization of chicks *in vivo*. Pretreatment with LG2055 significantly reduced adhesion to and invasion of a human epithelial cell line, Intestine 407, by *C. jejuni* 81–176. Methanol (MeOH)-fixed LG2055 also reduced infection by *C. jejuni* 81–176. However, proteinase K (ProK)-treated LG2055 eliminated the inhibitory effects. Moreover, LG2055 co-aggregated with *C. jejuni* 81–176. ProK treatment prevented this co-aggregation, indicating that the co-aggregation phenotype mediated by the proteinaceous cell-surface components of LG2055 is important for reducing *C. jejuni* 81–176 adhesion and invasion. In an *in vivo* assay, oral doses of LG2055 were administered to chicks daily for 14 days after oral inoculation with *C. jejuni* 81–176. At 14 days post-inoculation, chicks treated with LG2055 had significantly reduced cecum colonization by *C. jejuni*. Reduction in the number of *C. jejuni* 81–176 cells adhering to and internalized by human epithelial cells demonstrated that LG2055 is an organism that effectively and competitively excludes *C. jejuni* 81–176. In addition, the results of the chick colonization assay suggest that treatment with LG2055 could be useful in suppressing *C. jejuni* colonization of the chicks at early growth stages.

## Introduction

*Campylobacter jejuni* infection (campylobacteriosis), which leads to *g*astroenteritis in humans, is one of the leading causes of enteric bacterial infections [1]–[3]. *C. jejuni* colonization in the ileum and colon can interfere with normal functions of the human gastrointestinal (GI) tract. *C. jejuni* infection is characterized by fever, abdominal cramps, and diarrhea [1], [4], [5]. Epithelial cell adhesion and invasion of the GI tract by the pathogen play important roles in the pathogenesis of the disease and are associated with other well defined disease traits, including induction of cell death and disruption of mucosal barrier function [2], [4]–[8]. Therefore, preventing *C. jejuni* adhesion to and invasion of human epithelial cells is critical for reducing campylobacteriosis.

Chickens are a natural host for *Campylobacter* species, in particular, *C. jejuni*. One study detected considerable *Campylobacter* contamination in chicken carcasses, with an average prevalence of over 60% [9]. Chicken meat products are the main source of campylobacteriosis in humans [10], [11]. Thus, reducing *C. jejuni* colonization of the chick intestinal tract has been proposed as a strategy to reduce the disease burden in humans [4], [12].

Probiotic bacteria, such as *Lactobacillus* strains, can competitively inhibit *C. jejuni* colonization and infection [13]. Competitive inhibition of *C. jejuni* by *Lactobacillus* may occur through several key mechanisms, including competition for attachment sites [14], co-aggregation with the pathogen [15], [16], antimicrobial compound production [17]–[19] hydrogen peroxide production [20], and lactic acid production [21]. *Lactobacillus gasseri* SBT2055 (LG2055), a strain isolated from human feces, exhibits good fermentative properties and strong resistance to artificial gastric and bowel fluid [22]; furthermore, this strain can colonize the GI tract of humans and of mice [22], [23]. Moreover, LG2055 in the human intestine significantly decreases the number of fecal staphylococci [22]. LG2055 administration is effective for protection against influenza A virus infection in mice [24]. However, despite these findings, the inhibitory effect of LG2055 on *C. jejuni* infection is unknown at present.

The *C. jejuni* 81–176 strain, originally isolated during an outbreak of campylobacteriosis [25], can persistently colonize the GI tract of chicks [25], [26] and invade human epithelial cells [27]. In the present work, we evaluated the ability of LG2055 to inhibit adhesion and invasion of *C. jejuni* 81–176 *in vitro* as well as its ability to inhibit *C. jejuni* 81–176 colonization of chicks *in vivo*. We also sought to clarify the inhibitory mechanisms of LG2055.

## Materials and Methods

### Bacterial strains and growth conditions

*C. jejuni* 81–176 was cultured at 37°C under microaerophilic conditions on Mueller–Hinton (MH) agar plates (BD Difco, NJ, USA) for 48 h. The bacteria were transferred to a biphasic medium in 25-cm^2^ tissue culture flasks (Techno Plastic Products, Switzerland), containing MH agar and 5 mL of MH broth, and then cultured to an OD_600_ of 0.5. The *L. gasseri* SBT2055 strain (LG2055) in this study was provided by Megmilk Snow Brand Co., Ltd. (Saitama, Japan) and was cultured on de Man-Rogosa-Sharpe (MRS) agar plates (BD Difco) at 37°C for 24 h under anaerobic conditions. The bacteria were transferred to MRS broth and cultured to an OD_600_ of 2.0.

### Epithelial cell line and culture conditions

The epithelial cell line Intestine 407 (Int407) was obtained from the American Type Culture Collection (VA, USA). The cells were cultured in RPMI 1640 medium (Gibco, NY, USA) with 10% (v/v) fetal bovine serum (FBS), 10 U/mL penicillin (Gibco), and 10 µg/mL streptomycin (Gibco). Cells were maintained in 25-cm^2^ flasks and then seeded onto 12-well plates (37°C, 5% CO_2_). Cells were grown for a minimum of 10 days, and the medium was changed every 3 days. Antibiotics and FBS were removed from the cells at least 24 h before the infection assays.

### Anti-*C. jejuni* activity

Anti-*Campylobacter* activity was evaluated by the agar well diffusion method [21] with several modifications. Overnight culture supernatants of LG2055 were collected, and either boiled for 6 min, neutralized to pH 7 with 6 N NaOH, or left untreated. Supernatants were subsequently filter sterilized (0.22-µm filter). MH agar plates were overlaid with 15 mL of molten MH soft agar (0.75%) inoculated with 300 µL of *C. jejuni* 81–176 cultures, standardized to an OD_600_ of 1.0 in MH broth. Wells of 5-mm diameter were cut into agar plates, and 25 µL of the LG2055 supernatant was added to each well. After 24 h incubation under microaerophilic conditions at 37°C, the diameter of the zone of inhibition around each well was measured.

### Tissue culture adhesion and invasion assays

Adhesion and invasion assays were performed as described previously, with some modifications [14]. Confluent monolayers of Int407 cells (approx. 2×10^5^ cells/well) were washed twice with phosphate-buffered saline (PBS; pH 7.4). Bacterial cells were resuspended in 1 mL of RPMI 1640 medium to a multiplicity of infection (MOI) of 0.1∶1 to 1000∶1 for *C. jejuni* 81–176 and a ratio of interaction (ROI) of 0.1∶1 to 1000∶1 for LG2055. For the adhesion assay, bacterial cells were then added to each well and incubated at 37°C in 5% CO_2_ for 4 h. Subsequently, the wells were washed three times with PBS to remove non-adherent bacteria and incubated with 500 µL of 0.1% (v/v) Triton X-100 in PBS. Serial dilutions of the bacteria were plated onto MH or MRS agar plates and cultured under microaerophilic or anaerobic conditions at 37°C for 24 h, respectively. After incubation, colony-forming units (CFU) were counted.

For the invasion assay, bacterial cells were added to each well with Int407 monolayers, and the plates were incubated at 37°C in 5% CO_2_ for 4 h. The wells were then washed three times with PBS, fresh RPMI 1640 medium containing 100 µg/mL gentamicin (Wako, Osaka, Japan) was added, because gentamicin (100 µg/mL) effectively kills all extracellular bacteria [28], and then the samples were incubated further for 2 h. The cells were then washed three times with PBS. The count for the invasive bacteria was performed, as described above, by using MH agar plates.

In the assay for inhibition of adhesion and invasion, LG2055 cells (ROI of 10∶1, 100∶1, and 500∶1) were added to the Int407 monolayers 1 h before inoculation with *C. jejuni* 81–176 (MOI of 100∶1 for invasion, 500∶1 for adhesion). The *C. jejuni* 81–176 suspension was added to the Int407 monolayers without washing off the LG2055 cells, and the mixture was incubated at 37°C in 5% CO_2_ for 4 h. Subsequently, the wells were washed three times with PBS to remove non-adherent bacteria. The assay to quantify adhesion and invasion was the same as described above.

### Methanol (MeOH) fixation and proteinase K (ProK) treatment of LG2055

MeOH fixation and ProK treatment of LG2055 were performed as described previously [29], with some modifications. Briefly, LG2055 cells were resuspended in PBS to a concentration of 1×10^8^ CFU/mL (ROI of 500∶1). The bacterial cell suspensions were treated with an equal volume of ice-cold MeOH for 10 min or with 0.2 mL of RPMI 1640 containing 1 mg of ProK for 2 h at 37°C. Bacterial suspensions were centrifuged (6,000×*g*, 5 min, for 4°C), and the bacterial pellet was resuspended in 1 mL of RPMI 1640. Microscopic visualization of the gram-stained samples confirmed that the lactobacilli were intact after MeOH fixation and ProK treatment. The adhesion and invasion tests were performed as described above.

### Self- and co-aggregation assays

The ability of bacteria to self- and co-aggregate was assessed according to a previously described method [16] with some modifications. Bacterial cells were suspended in 3 mL of PBS at a concentration of 1×10^8^ CFU/mL. To determine self-aggregation, bacterial suspensions were statically incubated in aliquots at 25°C for 3 h. To determine the ability of bacteria to co-aggregate, equal volumes of LG2055 or ProK-treated LG2055 and *C. jejuni* 81–176 suspensions were mixed, and the mixtures were statically incubated at 25°C for 3 h.

To further characterize the self- and co-aggregation, surface plasmon resonance (SPR) studies using a Biacore X instrument (GE Healthcare, NJ, USA) were performed. LG2055 or ProK-treated LG2055 were immobilized on a CM5 dextran sensor chip with 5650 and 5820 resonance units (RUs), respectively, by using amine-coupling reagents (GE Healthcare). The analytes, which were bacterial cells, were suspended in PBS containing 0.005% surfactant P20 (pH 7.4) to a concentration of 1×10^8^ CFU/mL. Multicycle experiments were performed as described [30], with an analyte flow rate of 3 µL/min for 10 min at 25°C per cycle.

### Chick colonization assay

Chicken colonization studies were performed as described previously [31]. White leghorn chicken eggs were supplied by a commercial farm (Koiwai Farm, Ltd., Iwate, Japan) and maintained in an egg incubator until the chicks hatched. Approx. 24 h after hatching, chicks were randomly assigned to two groups. Bacterial cells were washed and resuspended in ice-cold PBS prior to inoculation. All birds were administered 1×10^6^ CFU of *C. jejuni* 81–176 in a 100-µL suspension by oral gavage. Twenty-four hours after oral gavage, LG2055 (1×10^8^ CFU in 100 µL) was orally administered daily to one group of *C. jejuni*-inoculated birds. PBS was administered to the other group of birds as a control. Chicks were sacrificed at 14 days post-inoculation, and the cecal contents were diluted in ice-cold PBS to 0.1 g/mL. Ten-fold serial dilutions of each sample were prepared and then plated on MH agar containing trimethoprim and cefoperazone to select for *C. jejuni*. MH agar plates were incubated under microaerophilic conditions at 37°C for 48 h. Cecal pH was measured with a compact pH meter (Model B-212; Horiba, Kyoto, Japan). The chicken experiments were performed at the Department of Animal Science, School of Veterinary Medicine, Kitasato University. All animal experiments were performed using protocols approved by the Institutional Animal Care and Use Committee at Kitasato University.

### Quantification of bacterial cells by real-time PCR

Quantification of bacterial cells by real-time PCR using 16S rDNA gene-specific primers was performed as described previously [32]. DNA was extracted from Int407 cells infected with bacterial cells and from chick cecal contents using an InstaGene Matrix (Bio-Rad Laboratories, CA, USA) or QIAamp DNA Stool Mini Kit (Qiagen, Hilden, Germany), respectively. Quantitative PCR was performed on a StepOnePlus Real-Time PCR System (Applied Biosystems, CA, USA). Duplicate 10 µL PCR reactions were carried out using Power SYBR Green Master Mix (Applied Biosystems) according to the manufacturer's instructions. Bacterial primers (F_eub: 5′-TCCTACGGGAGGCAGCAGT-3′, R_eub: 5′-GGACTACCAGGGTATCTAATCCTGTT-3′) [33], *Lactobacillus* spp. primers (LactoF: 5′-TGGAAACAGRTGCTAATACCG-3′, LactoR: 5′-GTCCATTGTGGAAGATTCCC-3′) [34], and *L. gasseri*-specific primers (LactoF, Lgass R: 5′-CAGTTACTACCTCTATCTTTCTTCACTAC-3′) [34] were used at final concentrations of 300, 200, and 400 nM, respectively. The reaction conditions were as follows: 95°C for 10 min followed by 40 cycles at 95°C for 15 s, annealing at the optimal temperature [33], [34] for 15 s, and elongation at 72°C for 20 s. The DNA samples extracted from *Escherichia coli* K12 (for total bacteria), *L. reuteri* JCM1112^T^ (for *Lactobacillus* spp.), and LG2055 (for *L. gasseri* strains) were used as real-time PCR standards. Standard bacterial DNA was prepared from a known number of bacterial cells (i.e., the number of CFU) and was found to be linear over the range of DNA concentrations from approx. 10^2^–10^8^ cells per PCR mixture. Bacteria were quantified using the standard-curve method, and all reactions were performed in triplicate in three independent experiments.

### Histochemical analysis

Cecal tissues of chicks (14 days old, 4 birds/group) were fixed without washing in 4% paraformaldehyde phosphate buffer solution (Wako) for 4 h at 25°C. Paraffin-embedded tissues were cut into 3-µm sections. After de-waxing in xylene and rinsing in alcohol, antigen retrieval was performed by placing the slides in 50 mM citrate buffer (pH 6.0) in an autoclave for 10 min at 110°C. Sections were then incubated with 5% (w/v) bovine serum albumin (BSA)–PBS solution for 3 h. Biotin-conjugated anti-*C. jejuni* antibody (1∶300 dilution; Fitzgerald Industries, MA, USA) was resuspended in 2% BSA–PBS solution and incubated for 12 h at 4°C. After washing, Cy3-conjugated streptavidin (1∶1000; GE Healthcare) was added and incubated for 40 min. Counterstaining was performed with 4′,6-diamidino-2-phenylindole (DAPI; Wako). The sections were briefly washed with PBS and mounted. Pictures were obtained with an Olympus BX53 Microscope (Tokyo, Japan).

### Statistical analyses

Prism 6 (GraphPad Software) was used for all statistical analyses. Significant differences were determined by using one-way analysis of variance (ANOVA) with *post hoc* Dunnett's test, or Mann–Whitney U test. “n” represents the number of individual experiments. Differences with *p* values less than 0.05 were considered significant.

## Results

### Capacity for invasion of and adhesion to Int407 cells

**Figure 1:**
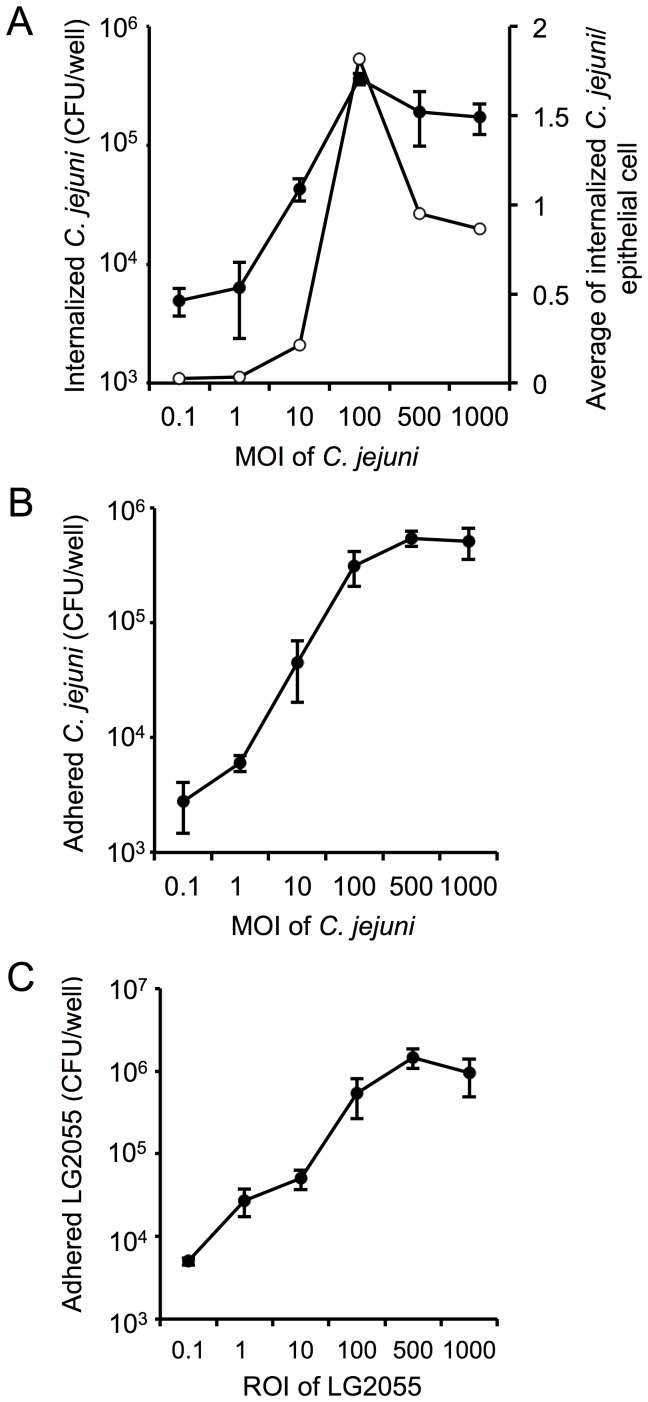
Invasion and adhesion by *C. jejuni* 81–176 or adhesion by LG2055 to Int407 cells. Effect of varying MOI on efficiency of *C. jejuni* 81–176 invasion (**A**) and adhesion (**B**). Int407 cells were infected with *C. jejuni* 81–176. The invasion and adhesion assays were conducted as described in materials and methods. Results are presented as the mean CFU per well (•), and the resulting average number of internalized *C. jejuni* 81–176 per epithelial cell (○). (**C**) Effect of varying ROI on LG2055 adhesion efficiency. Int407 cells were treated with LG2055. The adhesion assay was conducted as described in materials and methods. Results are presented as the mean CFU per well. All experimental error bars indicate standard deviations (n = 3).

We tested the invasive properties of *C. jejuni* 81–176 *in vitro*. *C. jejuni* 81–176 was co-cultured with Int407 cells at MOIs ranging from 0.1∶1 to 1000∶1. *C. jejuni* 81–176 was internalized by Int407 cells in a dose-dependent manner (MOI≤100∶1) (Figure 1A). We quantified the average number of internalized bacterial cells per Int407 cell by dividing the number of internalized bacteria by the total number of epithelial cells per well at each MOI tested. The highest possible number of internalized bacterial cells, which we observed at an MOI of 100∶1, was approx. 1.8 bacterial cells per epithelial cell (Figure 1A). This level of internalization is similar to the results of previous studies [35]. For comparison, the adhesive properties of *C. jejuni* 81–176 were also assessed. *C. jejuni* 81–176 adhered dose-dependently to Int407 cells, with the highest number of adherent bacteria at an MOI of 500∶1 (Figure 1B).

We next tested the adhesion abilities of LG2055 *in vitro*. LG2055 was co-cultured with Int407 cells at ROIs ranging from 0.1∶1 to 1000∶1. LG2055 also adhered to Int407 cells in a dose-dependent manner (Figure 1C), with the highest number of adherent bacteria at an ROI of 500∶1.

### Inhibition of *C. jejuni* 81–176 adhesion and invasion by LG2055

**Figure 2:**
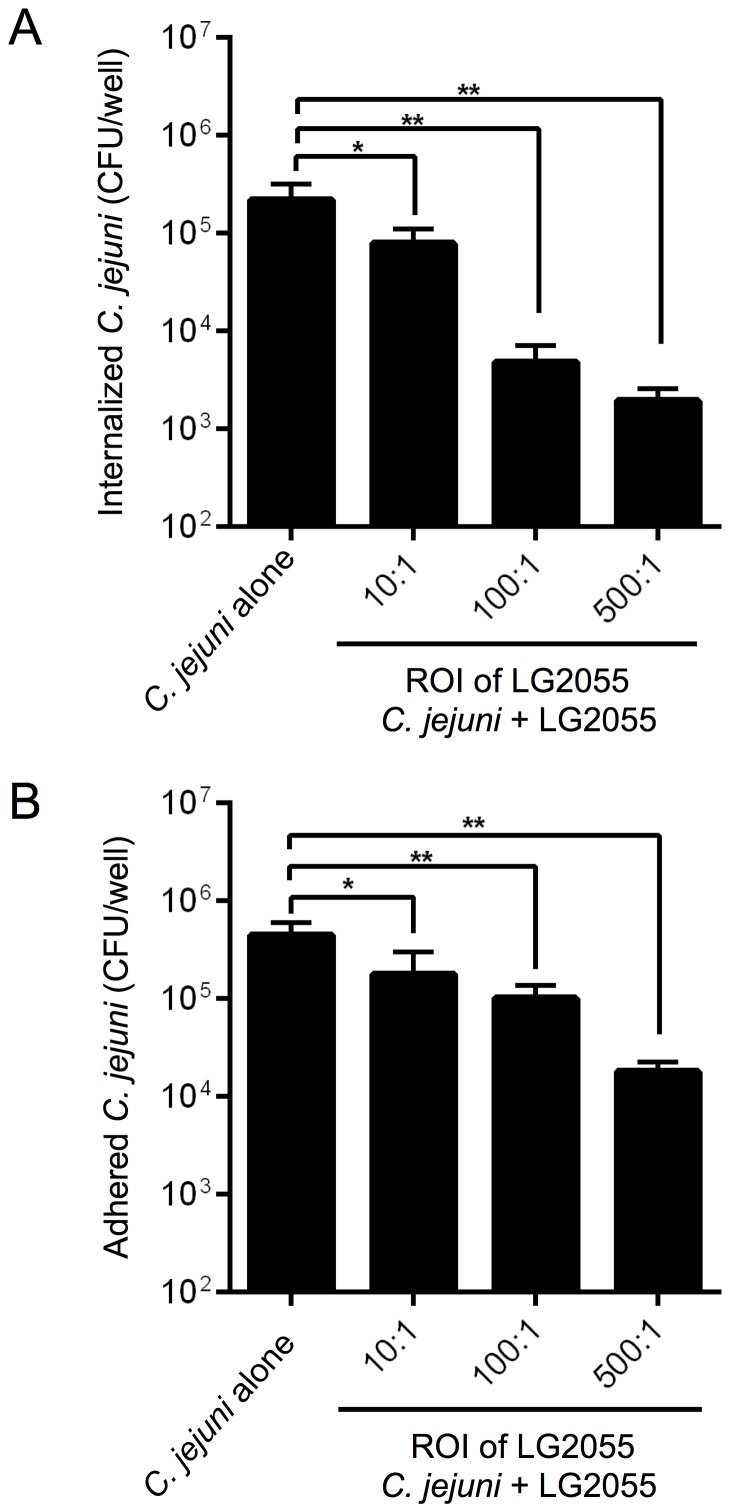
Inhibition of *C. jejuni* 81–176 invasion and adhesion to Int407 cells by LG2055. Assays for *L. gasseri*-mediated inhibition of invasion (**A**) and adhesion (**B**) were performed by pre-incubation of Int407 cells with LG2055 at the ROI of 10∶1, 100∶1, or 500∶1 followed by the addition of *C. jejuni* 81–176 at the MOI of 100∶1 (for invasion), or 500∶1 (for adhesion). Results are presented as the mean CFU per well. The asterisks indicate that the number of *C. jejuni* 81–176 was statistically different (*p<0.05, **p<0.01) than that of the untreated groups, as determined by one-way ANOVA with the *post hoc* Dunnett's test. Error bars indicate standard deviations (n = 5).

To examine whether LG2055 competitively inhibited the adhesion and invasion of *C. jejuni* 81–176 *in vitro*, Int407 cells were pre-incubated with LG2055 at an ROI of 10∶1, 100∶1, or 500∶1, and then Int407 cells were incubated with *C. jejuni* 81–176 at an MOI of 100∶1 (for invasion), or 500∶1 (for adhesion), respectively. Pre-incubation with LG2055 resulted in approx. 3- to 100-fold decrease in the number of internalized bacteria (Figure 2A) and approx. 2.5- to 25-fold decrease in adherent bacteria (Figure 2B).

### Characterization of *C. jejuni* 81–176 inhibition effects by LG2055

**Figure 3:**
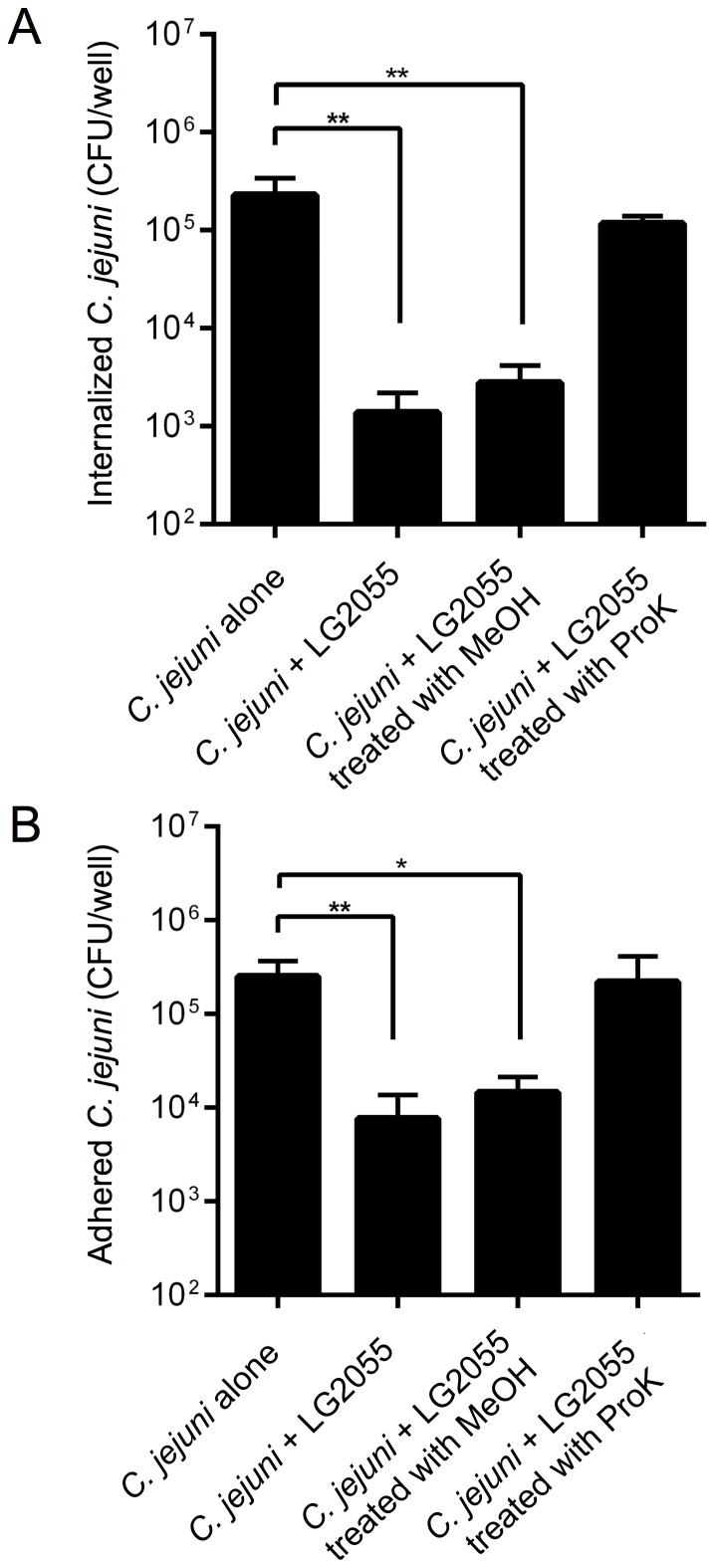
Inhibition of *C. jejuni* 81–176 invasion and adhesion to Int407 cells by MeOH-fixed or ProK-treated LG2055. LG2055 cells were treated with MeOH or ProK prior to infection of Int407 cells. Assays for LG2055-mediated inhibition of invasion (**A**) and adhesion (**B**) were performed by pre-incubation of Int407 cells with treated or untreated LG2055 at the ROI of 500∶1 followed by the addition of *C. jejuni* 81–176 at the MOI of 100∶1 (for invasion), or 500∶1 (for adhesion). Results are presented as the mean CFU per well. The asterisks indicate that the number of *C. jejuni* 81–176 was statistically different (*p<0.05, **p<0.01) than that of the untreated groups, as determined by one-way ANOVA with the *post hoc* Dunnett's test. Error bars indicate standard deviations (n = 5).

Surface components of probiotic bacteria have been shown as potential reactants with pathogens [13], [15], [16], [29], [36]. To evaluate *C. jejuni* 81–176 inhibition caused by LG2055, we focused on the proteinaceous cell surface components of LG2055 that were involved in inhibition by examining the effects of ProK-treated LG2055 on *C. jejuni* 81–176 invasion and adhesion. ProK treatment eliminated LG2055-mediated inhibition of *C. jejuni* 81–176 invasion (Figure 3A) and adhesion (Figure 3B). By contrast, in an agar diffusion assay, untreated and heat-treated LG2055 supernatants greatly inhibited *C. jejuni* 81–176 growth (Figure S1). Therefore, we next examined whether the metabolically active components of LG2055 were involved in *C. jejuni* 81–176 inhibition. MeOH-fixed LG2055 significantly decreased invasion by approx. 83-fold (Figure 3A) and significantly reduced adhesion by 17-fold (Figure 3B), similar to the levels of inhibition observed with the intact cells.

**Figure 4:**
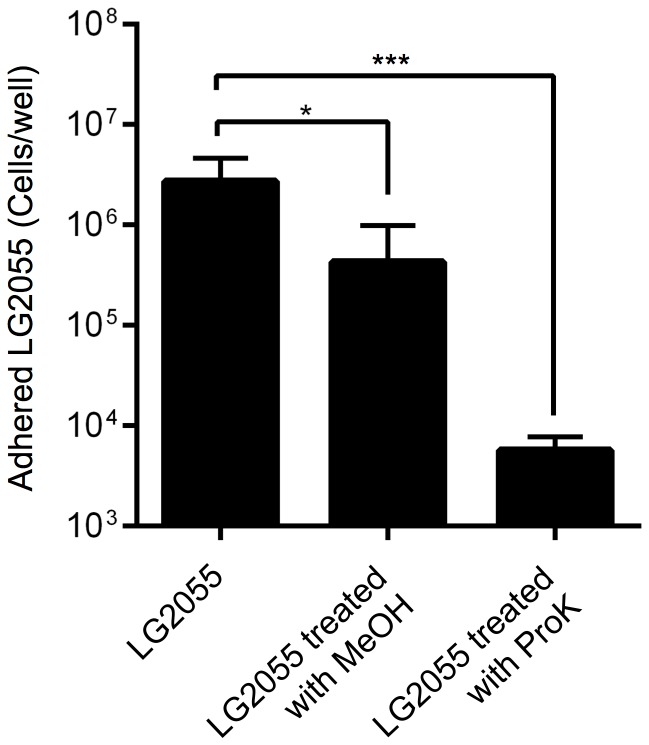
Quantification of adhesion of MeOH-fixed or ProK-treated LG2055 strains to Int407 cells in an adhesion inhibition assay. The numbers of *L. gasseri* strains were quantified by real-time PCR using *L. gasseri*-specific primers. The asterisks indicate that the number of LG2055 was statistically different (*p<0.05, ***p<0.001) than that of the untreated LG2055, as determined by one-way ANOVA with the *post hoc* Dunnett's test. Error bars indicate standard deviations (n = 5).

The adhesion property of LG2055 in an adhesion inhibition assay was assessed using quantitative real-time PCR. The adhesion capacity was significantly reduced by treating the cells with MeOH (*p*<0.05) and ProK (*p*<0.001), compared with that of untreated LG2055 cells (Figure 4). In particular, the adhesion of ProK-treated LG2055 cells was greatly reduced by approx. 470-fold. These results showed that the inhibitory factor is a protein and/or cell-surface component.

### Characterization of LG2055 co-aggregation with *C. jejuni* 81–176

**Figure 5:**
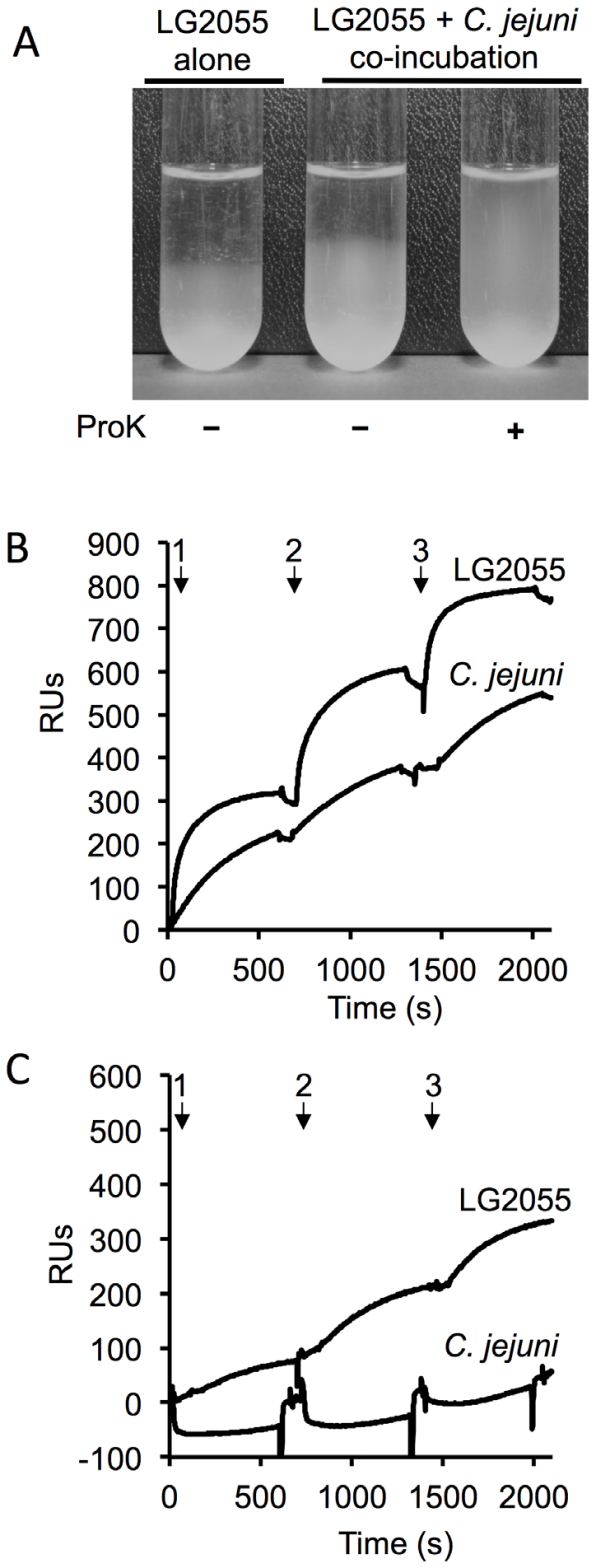
Co-aggregation of LG2055 and *C. jejuni* 81–176. (**A**) The left tube shows self-aggregation of LG2055, the center tube shows co-aggregation of LG2055 with *C. jejuni* 81–176, and the right tube shows co-aggregation of ProK-treated LG2055 with *C. jejuni* 81–176. (**B, C**) Interaction of bacterial cells by SPR analysis. The SPR biosensors presented the following amounts of immobilized ligands: 5650 RUs, LG2055 (**B**); and 5820 RUs, ProK-treated LG2055 (**C**). The live bacterial cells were suspended in running buffer and injected as an analyte during each cycle. The SPR analysis was conducted as described in materials and methods. Arrows indicate sample inject point.

Co-aggregation, a probiotic interaction with pathogenic microorganisms, may lead to the formation of a barrier that prevents colonization by the pathogens [36]–[38], which is usually facilitated by surface-exposed proteinaceous components [15], [16]. Aggregation of LG2055 was examined by statically incubating bacterial suspensions over a period of 3 h. The strain exhibited a strong self-aggregation phenotype (Figure 5A, left tube). Co-incubation of LG2055 with *C. jejuni* 81–176 also resulted in a similar aggregation phenotype (Figure 5A, middle tube), while ProK pretreatment of LG2055 abolished co-aggregation (Figure 5A, right tube).

To further characterize the aggregation, we examined the interaction between bacterial cells by SPR analysis. We measured the ability of live LG2055 and *C. jejuni* 81–176 cells to bind LG2055 cells immobilized on a biosensor. Both bacteria bound to the immobilized cells during the three-cycle experiment (Figure 5B). Moreover, in a three-cycle binding experiment using immobilized ProK-treated LG2055, live *C. jejuni* 81–176 cells did not bind to the bacterial ligand (Figure 5C). In contrast, live LG2055 cells showed moderate binding to the ligand.

### Inhibition of *C. jejuni* colonization in chicks by LG2055

**Figure 6:**
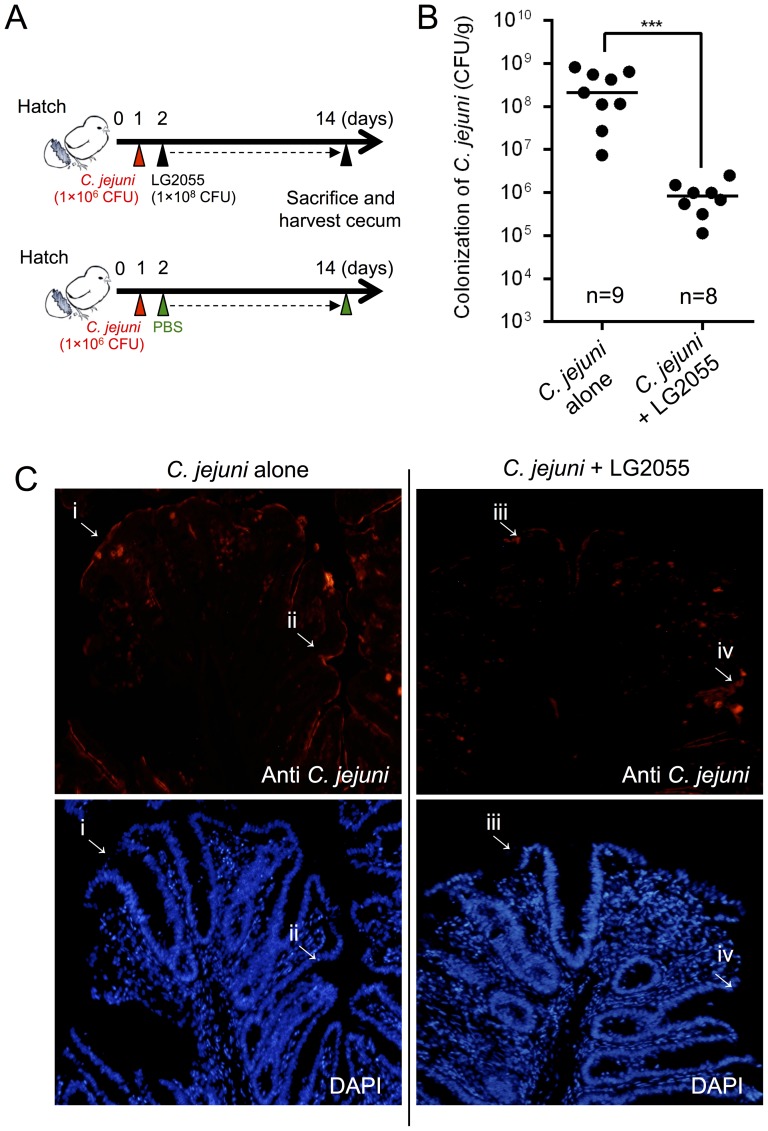
Colonization of *C. jejuni* in the chick cecum. (**A**) Twenty-four hours after hatching, chicks were inoculated orally. LG2055 was administered orally to one group of *C. jejuni* 81–176-inoculated birds daily. PBS was administered to the other group of birds as a control. (**B**) Chicks were sacrificed at days 14 post-inoculation, and colonization with *C. jejuni* was quantified by counting CFU using MH agar plates. The bar indicates the median CFU for each group, which was determined using all birds within the group. The LG2055-administered group of birds exhibited significant suppression of *C. jejuni* colonization (***p<0.001), as determined by the Mann-Whitney U test. “n” indicates the number of chicks in each group of 9 that were colonized with *C. jejuni* (limit of detection, 10^3^ CFU/g of cecal contents). (**C**) Immunohistochemical staining of chick cecal tissue at 14 days post-inoculation. Cecal sections were probed with anti-*Campylobacter* antiserum or stained with DAPI. Fluorescence microscopy shows *C. jejuni* colonization (arrows: i–iv) over the mucosal surface of the cecal tissue at 100× magnification.

The inhibitory effect of LG2055 on *C. jejuni* colonization in chicks was examined. Approx. 24 h after hatching, chicks were pre-inoculated orally with *C. jejuni* 81–176, and then LG2055 was administered daily (Figure 6A). *C. jejuni* cells were counted in the untreated control group at 14 days post-inoculation; the median number of *C. jejuni* in the ceca increased to 2.0×10^8^ CFU/g of cecal content at 14 days post-inoculation (Figure 6B). In contrast, the median level of *C. jejuni* colonization in chicks treated with LG2055 significantly decreased by approx. 250-fold, compared with the levels of the untreated control group. In addition, immunohistochemical staining of chick cecal tissue and fluorescence microscopy showed *C. jejuni* colonization over the mucosal surface of the cecal tissue (Figure 6C, arrows i–iv). The fluorescence signal of *C. jejuni* was lower in chicks treated with LG2055 (Figure 6C, arrows iii and iv) than in the chicks from the untreated control group (Figure 6C, arrows i and ii).

**Figure 7:**
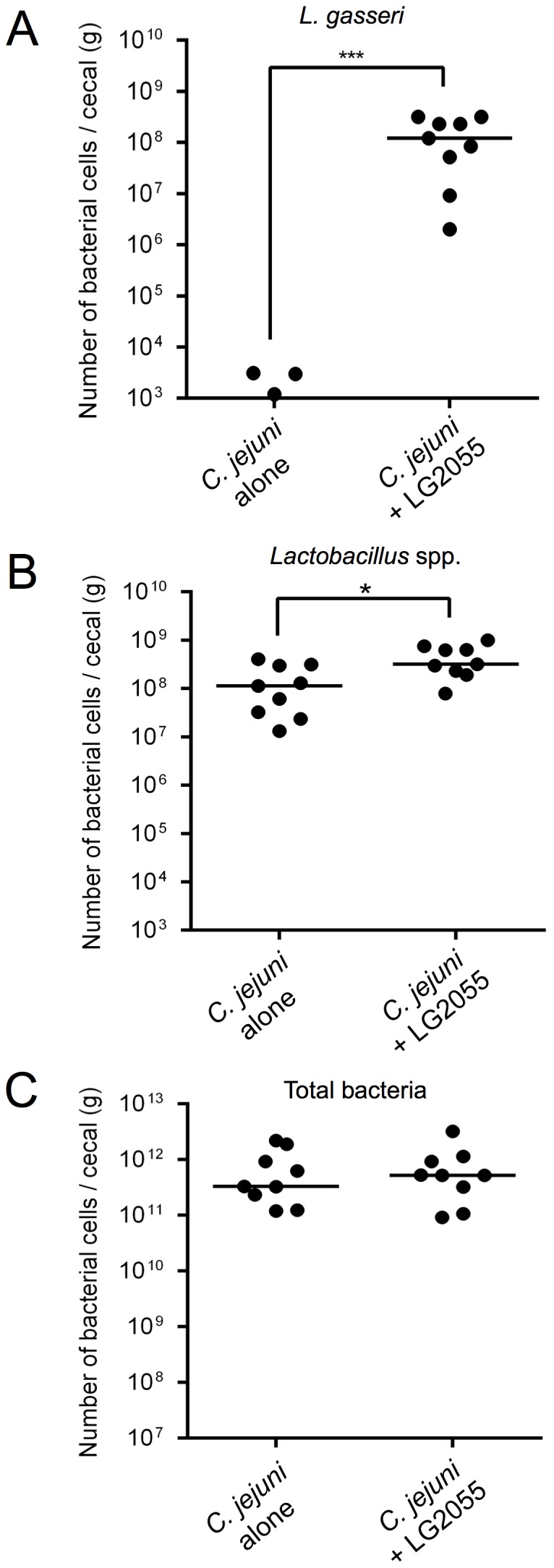
Quantification of gut bacteria at 14 days post-inoculation. The numbers of *L. gasseri* (**A**), *Lactobacillus* spp. (**B**), and total bacteria (**C**) in cecal samples were quantified by real-time PCR using 16S rDNA gene-specific primers. The LG2055-administered group of birds showed significantly increased levels of *L. gasseri* and *Lactobacillus* spp. (*p<0.05, ***p<0.001), as determined by the Mann-Whitney U test.

Quantitative real-time PCR analysis of 16S rDNA with species- or genus-specific primers was used to quantify cecal bacteria at 14 days post-inoculation. Colonization by *L. gasseri* and *Lactobacillus* spp. in the group treated with LG2055 was significantly higher than in the untreated control group, but colonization did not alter the total bacteria in the ceca (Figure 7A–C). The number of *L. gasseri* cells in the ceca of the untreated group was below the limit of detection by real-time PCR (<10^3^ cells/g) (Figure 7A). There were no significant differences in cecal pH values (Figure S2) and body weight. In addition, both groups of birds displayed similar appetites during the experiment.

## Discussion

LG2055 possesses a variety of probiotic activities and roles, including production of bacteriocin [39], inducing suppression of the number of fecal staphylococci [22], protection against influenza A virus infection [24], regulating adipose tissue growth in rats [40], and exerting an anti-obesity effect in humans [41]. Campylobacteriosis, the most commonly reported form of food-borne gastroenteritis, is mainly caused by the zoonotic pathogen *C. jejuni* [3], [5], [10], [12]. We have now demonstrated that LG2055 can also reduce the adhesion and invasion of *C. jejuni in vitro* and can inhibit pathogen colonization of chicks *in vivo*. In this study, we characterized the effective competitive ability of LG2055 to exclude this pathogen, which may be useful for the prevention of campylobacteriosis.

Intestinal epithelial cells are the initial site for host interactions with *Campylobacter*. Int407 cell lines of human intestinal epithelium origin are considered one of the most appropriate models for studying *Campylobacter* infection [42]. In this study, we established the ability of LG2055 to reduce *C. jejuni* 81–176 adhesion to Int407 cells. Treatment with LG2055 resulted in greater than 2-log-fold reduction in *C. jejuni* 81–176 invasion of the epithelial cells. Previous papers reported a protective effect of probiotic strains on epithelial cells infected with *Campylobacter*. For example, *L. helveticus* R0052 adheres to human colonic T84 and Int407 cells, reducing *C. jejuni* invasion into these cells by 35%–55% following co-culture with probiotics [14]. However, comparison of previous results with our current results shows that LG2055 is more effective at inhibiting *C. jejuni* infection.

An anti-*Campylobacter* analysis showed that LG2055 inhibits *C. jejuni* 81–176 growth by means of an acidic component instead of a heat-labile component. Lactic acid produced by *L. crispatus* JCM5810 inhibits *C. jejuni* growth and colonization *in vitro* and *in vivo* [21]. Although acidity induced by LG2055 might affect *C. jejuni* 81–176 colonization, pretreatment with MeOH-fixed LG2055 reduced pathogen adhesion and invasion. In addition, ProK treatment of LG2055 eliminated the inhibitory effect. Taken together, these data suggest that inhibition of *C. jejuni* 81–176 invasion of and adhesion to epithelial cells might involve constitutive proteinaceous components on the exposed surface of LG2055, rather than soluble metabolically active components.

Furthermore, we showed that LG2055, but not ProK-treated LG2055, co-aggregated with *C. jejuni* 81–176. These results support our observations of the *in vitro* inhibition effects. Previous reports also demonstrated effective *in vitro* co-aggregation of *Campylobacter* strains with a surface component of *Lactobacillus* [15], [16]. Thus, we presumed that co-aggregation properties of LG2055 with *C. jejuni* 81–176 mediated by proteinaceous, cell-surface component(s) of LG2055 play a key play in preventing this pathogen infection. On the other hand, ProK treatment of LG2055 dramatically reduced the ability to adhere to Int407 cells. Competition with pathogens for adhesion and colonization of the mucosal surface are possible protective mechanisms employed by probiotics [14], [29], [36]. This competition suggests that the inhibition mechanisms against infection and colonization involve other distinct processes in addition to the co-aggregation phenotype.

In our *in vivo* model of *Campylobacter* colonization, we demonstrated that LG2055 was able to significantly reduce colonization in chicks at 14 days post-inoculation. Histochemical analysis also showed reduced adhesion of *C. jejuni* on the cecal mucosa of chicks treated with LG2055. A previous study demonstrated that most *Campylobacter* colonization occurs at an age of two to four weeks [43], which is probably delayed because of the presence of maternally derived antibodies inhibiting *Campylobacter* colonization in young chicks [44]. Moreover, once *Campylobacter* colonization is detected in chickens, the prevalence of *Campylobacter* colonization increases from 5%–95% within several days [45]. Therefore, our results imply that, although LG2055 cannot completely inhibit *C. jejuni* colonization in chicks, treatment with LG2055 could be useful in suppressing pathogen colonization of the chicks at early growth stages, thereby helping to prevent pathogen infection. On the other hand, a previous study indicated that there is a high risk of *Campylobacter* colonization in chicken throughout the feeding period [43]. Therefore, we will need perform more long-term chicken colonization experiments to further evaluate the *Campylobacter* inhibition effects caused by LG2055.

Inoculation with LG2055 increased the number of *Lactobacillus* spp. and *L. gasseri* cells, as detected by quantitative real-time PCR, and the LG2055 strain exhibited a strong self-aggregation phenotype *in vitro*. Self-aggregation of probiotic strains correlates with adhesion to mucous components and biofilm formation, which is a prerequisite for colonization of the GI tract [36], [46]–[50]. Taken together, our results show that LG2055 may be able to establish persistent colonization and may become a part of the dominant microbiota. Moreover, the effects of acid production alone do not explain the inhibitory effects of LG2055 on *C. jejuni* colonization, as cecal pH values changed minimally. Some probiotic treatments reduce colonization by *Campylobacter*. Specifically, certain *Lactobacillus* strains, which are employed by the poultry industry and produce bacteriocins against *Campylobacter*, efficiently reduce the colonization of this pathogen in chickens during commercial processing [17], [19]. The anti-colonization effect by LG2055 is not yet applicable for use in the poultry industry; however, the co-aggregation phenotype and (or) adhesion capacity are important factors that should be considered in designing competitive exclusion strategies to reduce pathogen loads in livestock.

In conclusion, we have demonstrated that LG2055 is an organism that effectively and competitively excludes *C. jejuni*. This exclusion is evidenced by the reduction in the number of *C. jejuni* cells adhering to and internalized by epithelial cells of humans and chicks. Data from our *in vitro* experiments indicate that the co-aggregation phenotype and/or adhesion mediated by proteinaceous surface component(s) of LG2055 might be responsible for the reduction in *C. jejuni* infection and colonization. Extracellular aggregation-promoting factors (APFs) and genes encoding them for several *Lactobacillus* species, including *L. gasseri*, have been characterized previously [51]. APFs are secreted proteins that are associated with a diverse number of functional roles, including self-aggregation [52], maintenance of cell shape [53], and adhesion [47], [49]. Thus, further analysis is required to confirm the role and involvement of proteinaceous cell-surface components such as APFs in the inhibitory properties of LG2055.

## Supporting Information

Figure S1 **Inhibition of** ***C. jejuni*** **81–176 by LG2055.** Anti-*Campylobacter* activity was assessed using spotted lactobacilli cell-free culture supernatant. Overnight LG2055 culture supernatants were collected and either heat-treated (boiled), neutralized with NaOH, or left untreated. The supernatants were added to MH agar plates seeded with *C. jejuni* and incubated for 24 h at 37°C.
(TIFF)

Figure S2 **The effect of cecal pH on administered LG2055.** The plot shows the mean pH for each group, which was determined using all birds within the group. Error bars indicate standard deviations. “n” indicates the number of chicks in each group.
(TIFF)
