# Supplementary figures and images for: Lactobacillus gasseri SBT2055 Reduces Infection by and Colonization of Campylobacter jejuni
Source: PLoS One. 2014 Sep 29;9(9):e108827. doi: 10.1371/journal.pone.0108827 (PMC4181664; doi:10.1371/journal.pone.0108827)

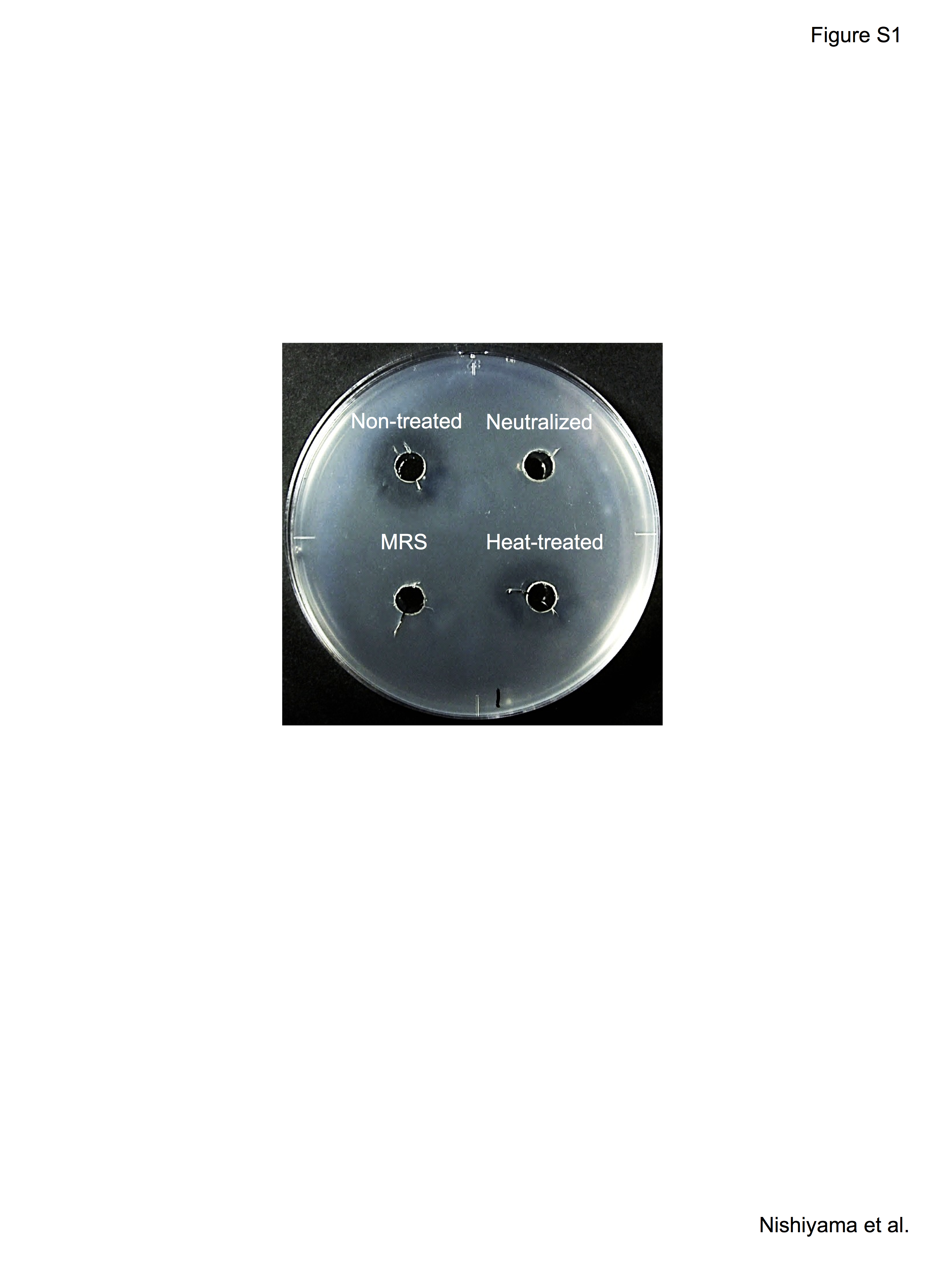

Supplement: Figure S1 — Inhibition of C. jejuni 81–176 by LG2055. Anti-Campylobacter activity was assessed using spotted lactobacilli cell-free culture supernatant. Overnight LG2055 culture supernatants were collected and either heat-treated (boiled), neutralized with NaOH, or left untreated. The supernatants were added to MH agar plates seeded with C. jejuni and incubated for 24 h at 37°C. (TIFF) [file pone.0108827.s001.tiff]

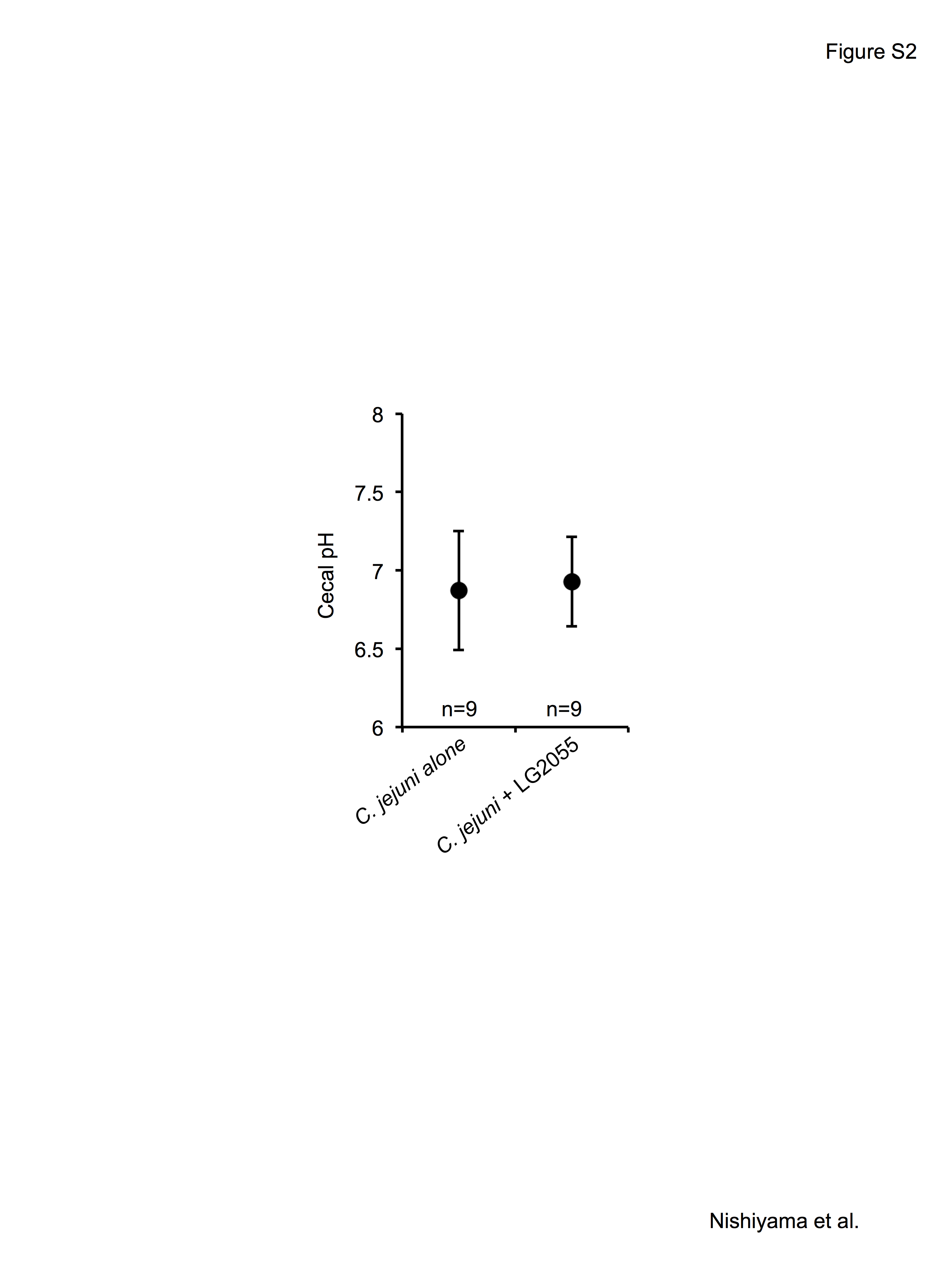

Supplement: Figure S2 — The effect of cecal pH on administered LG2055. The plot shows the mean pH for each group, which was determined using all birds within the group. Error bars indicate standard deviations. “n” indicates the number of chicks in each group. (TIFF) [file pone.0108827.s002.tiff]
